# Silent Slips: Overprescription of Benzodiazepines in Outpatient Psychiatry

**DOI:** 10.1192/bjo.2025.10682

**Published:** 2025-06-20

**Authors:** Asma Suleman, Anees Fatima, Nauman Mazhar, Ali Madeeh Hashmi

**Affiliations:** 1 Punjab Institute of Mental Health, Lahore, Lahore, Pakistan; 2 Mayo Hospital, Lahore, Pakistan

## Abstract

**Aims:** 1. To evaluate the prescribing patterns and duration of benzodiazepine use among psychiatric outpatients and their adherence to established guidelines.

2. To assess the prevalence of benzodiazepine dependence and the level of patient awareness regarding its associated risks.

3. To identify discrepancies between current prescribing practices and evidence-based guidelines, particularly in the treatment of Schizophrenia, Bipolar Affective Disorder, and Generalised Anxiety Disorder.

**Methods:** A retrospective review of online prescription records was conducted for 35 randomly selected patients from the outpatient department of the Punjab Institute of Mental Health, Lahore, during November 2024. The review adhered to the guidelines published by the Ministry of National Health Services, Regulations & Coordination, Pakistan (April 11, 2023), and the Maudsley Prescribing Guidelines in Psychiatry, 14th Edition.

The following parameters were analysed:

Demographic details and clinical diagnosis of the patients.

Benzodiazepine use, including the type of benzodiazepine prescribed and the duration of use.

Assessment of benzodiazepine dependence, using the Severity of Dependence Scale (SDS), with a score greater than 7 indicating dependence.

Evaluation of whether patients were informed about the potential risks of dependence prior to initiating benzodiazepine treatment.

**Results:** Demographics and diagnoses: Out of the 35 patients audited, 29 (82.9%) were male. Diagnoses included Schizophrenia (11 patients, 31.4%), Bipolar Affective Disorder (21 patients, 60%), and Generalized Anxiety Disorder (3 patients, 8.6%).

Benzodiazepine use: A total of 97.1% (34 patients) were prescribed benzodiazepines, with 85.7% (30 patients) on long-term use exceeding six months. Commonly prescribed benzodiazepines included clonazepam (2 mg), bromazepam (3 mg), and lorazepam (1 mg), often based on pharmacy stock availability rather than patient-specific indications.

Dependence and awareness: Dependence symptoms were identified in 25 patients (71.4%). 65.7% (23 patients) were not informed about the risks of dependence or proper duration of benzodiazepine use.

Guideline discrepancies: Contrary to established guidelines recommending benzodiazepines for short-term use (2–4 weeks), these medications were prescribed for prolonged periods without regular risk assessments for dependence.

**Conclusion:** The audit reveals an alarming trend of prolonged benzodiazepine use among psychiatric outpatients, predominantly males with schizophrenia and bipolar disorder. The prescribing practices observed starkly contrast with international guidelines, such as NICE and Maudsley, which emphasise short-term use (2–4 weeks) and the necessity of ongoing risk assessments for dependence.

The lack of patient education on the risks of benzodiazepines and the absence of consistent, evidence-based prescribing protocols further exacerbates the problem. This is particularly concerning given the high rates of dependence and the potential for severe withdrawal symptoms upon abrupt discontinuation.

To address these issues, it is critical to:

Develop and implement national and institutional guidelines for benzodiazepine prescribing in chronic psychiatric patients.

Regularly review patients on benzodiazepines to assess the need for continued use.

Educate both healthcare providers and patients on the risks of long-term benzodiazepine use, dependence, and withdrawal.

Encourage the use of alternative, non-pharmacological interventions for managing anxiety and agitation.

